# Medical Opinion and Movement

**Published:** 1907-10-26

**Authors:** 


### Medical Opinion and Movement.

An instructive experiment has been carried out by the Education Committee of Darwen, in Lancashire. A special class of thirty-eight boys and seven girls was formed and placed for six weeks under the instruction of Mr. W. G. Yearsley, with the object of curing the pupils of stammering. The methods adopted consisted in breathing exercises to strengthen and practise the respiratory muscles, vocal gymnastics, and combined vocal, breathing, and physical exercises. There were also exercises in reading aloud in company at a fixed rate by the metronome. During the last weeks the principles involved were explained to the children, and reading exercises were given for the pupils to practise at home. The results appear to be highly satisfactory. The stammering habit practically disappeared in almost all cases, and there was also a marked improvement in the respiratory capacity and general condition. Such a successful result should encourage further attempts at other centres, and if experience shows that a short course is sufficient the services of one skilled teacher could be shared by several centres. The treatment once started in this way might easily be carried on under the guidance of ordinary school teachers instructed in its principles. Stammering is a serious handicap in all walks in life, and the best chances of a cure are offered when the child is treated at an early age.

Under the munificent gift of £41,000 by Sir Ernest Cassel for the ophthalmic relief of the Egyptian poor, Mr. A. F. MacCallan was appointed ophthalmic surgeon and chief inspector in 1903, to organise a travelling ophthalmic hospital. An account of his work in Egypt was given at the Exeter meeting of the British Medical Association. Accompanied by his Egyptian assistants and staff, he first made a tour of the more important towns in the Delta, and the poor in need of treatment readily responded to the invitation to attend. The movement was so successful that a second travelling hospital was soon organised. Owing to representations by Mr. MacCallan the Government has adopted the policy of building hospitals in the large towns, one or more each year, as the finances permit, so that Mr. MacCallan hopes in the course of the next few years to have a special ophthalmic hospital in each of the fourteen provinces of Egypt. In the meantime Egyptian doctors are being trained in ophthalmic surgery, and they will be transferred to the hospitals as occasion arises. Mr. MacCallan is also striving after the introduction of a system of inspection and treatment of the eyes of scholars in the Government schools and the teaching of ophthalmic hygiene. In 1904 inspection of the pupils in the Government school at Damietta showed that 56.9 per cent. were affected with trachoma, 9.7 per cent. had eye affections other than trachoma, and 25.5 per cent. had gross corneal changes sufficient to cause deterioration of vision. These facts are sufficient to justify the energetic measures that Mr. MacCallan is advocating by making them his life's work to carry through.

There is a fashion in drugs as in other things, but the fluctuations of fashion chiefly affect the new synthetic preparations, which are incessantly put upon the market, and which generally enjoy a short-lived reputation. On the other hand, the old well-tried drugs retain the confidence of the profession, and undergo but little change in consumption. Dr. Grimbert has recently compared the consumption of drugs during the last ten years in the hospitals of Paris by reference to the books of the Pharmacie Centrale des Hôpitaux. He finds that the supply of the well-established remedies has remained constant for many years. Thus for the last forty years 200 kilogrammes of opium have been supplied to the hospitals, and similarly constant annual quantities of cinchona, bromide of potassium, salicylate of soda, subnitrate of bismuth, calomel, and several other preparations have been consumed. On the other hand, the use of poisonous antiseptics, such as carbolic acid and corrosive sublimate, has fallen off considerably, while the quantity of hydrogen peroxide has increased from 1,000 to 102,000 litres. It may be doubted whether this antiseptic has grown in favour to the same extent on this side. It is certainly a powerful germicide, and has the advantage of being at the same time non-poisonous and non-corrosive, but it can only be used fresh, and rapidly decomposes on keeping, in spite of all precautions. Among the new remedies pyramidon, aspirin, urotropin, protargol and veronal are at present in demand.

The last report of the Advisory Committee on Plague, appointed by the Secretary of State, the Royal Society, and the Lister Institute makes the connection between rats and the spread of the disease among mankind even more evident than has hitherto been supposed. The investigations and experiments that have been carried out strongly suggest, if they do not definitely prove, that the infection is transmitted from the rat to man by the flea. It is already well known that, although the disease spreads so rapidly among the inmates of infected dwellings and districts, medical men and others visiting and attending the sick escape with impunity. These and other facts point to some other source of infection than transmission of the virus from man to man, and infection from the rat by the flea accords in all respects with known conditions in regard to the spread of the disease. It seems highly probable, therefore, that this is not only a possible but the chief means of infection in plague epidemics. In view of the prejudice of the native in India against rat-killing, Lieut.-Colonel A. Buchanan, I.M.S., suggests that this object might be attained by encouraging the keeping of cats. By taking a cat census in the Amraoti district, and by comparing the percentage of cats to houses with the mortality from plague in over 1,000 different villages, it has been ascertained that a high percentage of cats is accompanied by a marked freedom from the disease. Traditions among the people are in favour of keeping cats, so that the custom could easily be fostered.

